# Occupational well‐being of the work community in social and health care education during the COVID‐19 pandemic—A cross‐sectional study

**DOI:** 10.1002/nop2.1658

**Published:** 2023-02-24

**Authors:** Anneli Vauhkonen, Kirsi Honkalampi, Marja Hult, Mika Hujo, Terhi Saaranen

**Affiliations:** ^1^ Department of Nursing Science, Faculty of Health Sciences University of Eastern Finland Kuopio Finland; ^2^ School of Educational Sciences and Psychology, Philosophical Faculty University of Eastern Finland Joensuu Finland; ^3^ School of Computing, Faculty of Science and Forestry University of Eastern Finland Kuopio Finland

**Keywords:** cross‐sectional study, educator, health care, occupational well‐being, work community

## Abstract

**Aim:**

To evaluate factors related to the occupational well‐being of social and health care educators' work communities during the COVID‐19 pandemic.

**Design:**

A cross‐sectional study was conducted among social and health care educators in Finland 2020.

**Methods:**

Data (*n* = 552) were collected through a questionnaire containing continuous, Likert scale and categorical variables. Descriptive, exploratory factor analysis and multiple regression modelling were used for analysing the data.

**Results:**

Educators regarded their work as meaningful and experienced collegiality. Age and work experience were related to experiences on work community subscales and community occupational well‐being. Personal occupational well‐being and activities promoting occupational well‐being on the community level were most related to experiences of the work community and its well‐being. The activities that promote occupational well‐being on the work community level should be emphasized.

## 1 INTRODUCTION

The rapid changes in working life, such as the ageing population, digitalization and the COVID‐19 pandemic, alter the needs for occupational well‐being development (European Agency for Safety and Health at Work [EU‐OSHA], 2018; Ministry of Social Affairs and Health, 2022) and are also reflected in the work of educators on national and international levels (Golnick & Ilves, 2022; Keener et al., 2021). A large group of social and health care educators are approaching retirement age (Education Statistics Finland, 2021; Ministry of Social Affairs and Health, 2022), and high workload and stressful work are resulting in a shortage of skilled educators (Owens, 2017a; Westphal et al., 2016). Even before the COVID‐19 pandemic, psychosocial risks, such as challenging customer situations and rushes, were related to the planning, organization and management of work, exposing work‐related stress (EU‐OSHA, 2018; EU‐OSHA, 2020). Further challenges in educators' work management and well‐being were caused by the transition to distance education as a result of the COVID‐19 pandemic in the spring of 2020 (Chan et al., 2021; Keener et al., 2021).

Social and health care educators play an important role in ensuring that future social and health care professionals are capable of providing high‐quality and human‐centred care and social services (Mikkonen et al., 2019; World Health Organization [WHO], 2016). Educators' occupational well‐being is related to students' welfare (Harding et al., 2019; Saaranen et al., 2015), the quality of teaching (Saaranen et al., 2015), work productivity (Alker et al., 2015) and job retention (Chambers Mack et al., 2019; Yedidia et al., 2014). A well‐functioning work community with good information policies and work organization (Vauhkonen et al., 2021), a positive work atmosphere and employee appreciation (Laine et al., 2018b) support employee's occupational well‐being. Occupational well‐being, especially about the work community perspective, remains understudied in social and health care education. It is therefore crucial to find evidence‐based knowledge about occupational well‐being on the work community level, particularly when social interactions are reduced. Based on the results, methods can be created to improve occupational well‐being in social and health care education on the regional, national and global levels.

## 2 THEORETICAL BACKGROUND

**FIGURE 1:**
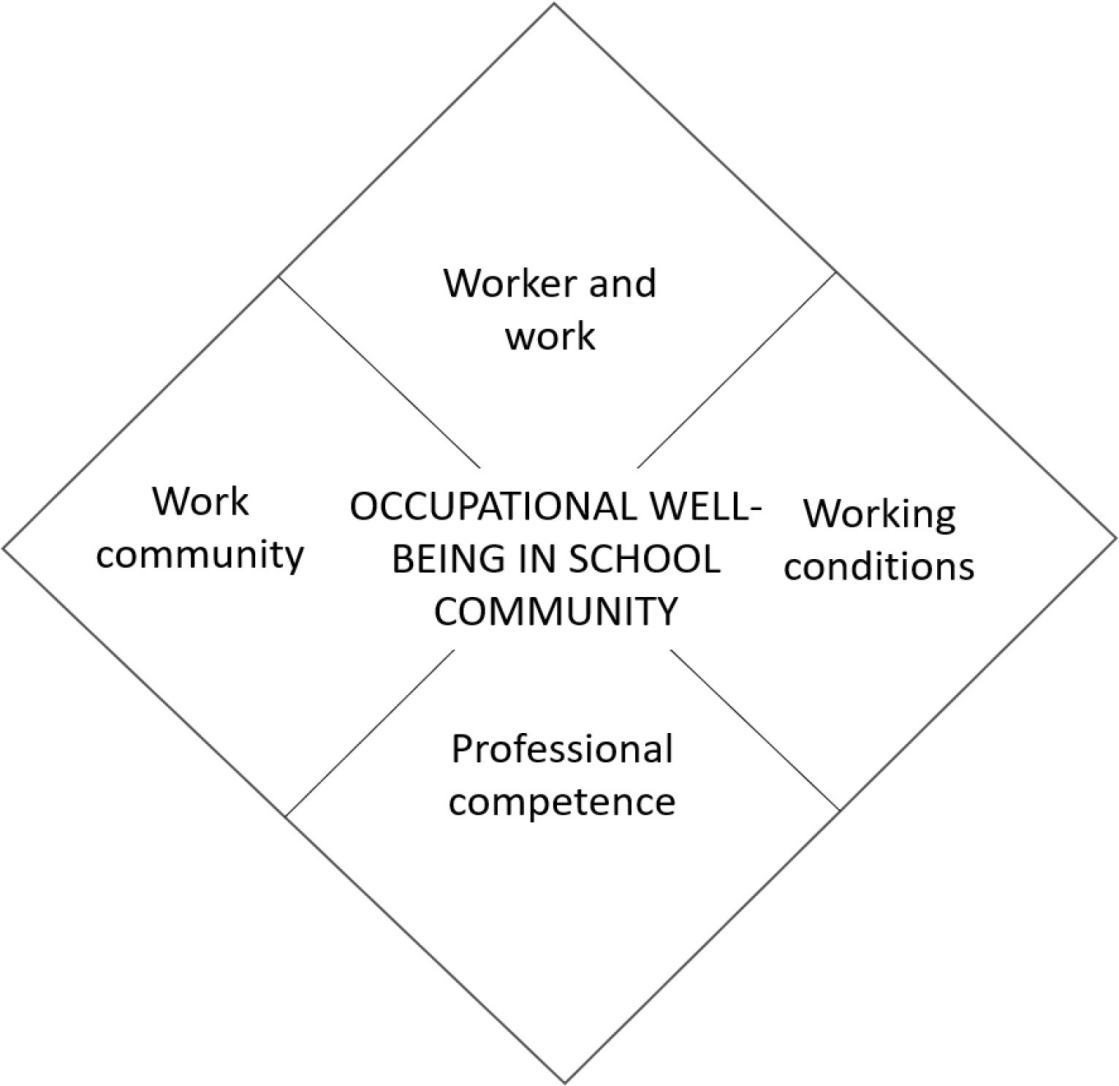
Content model for the promotion of School Community Staff's Occupational Well‐being (Saaranen et al., 2007, 2015).

In this research, occupational well‐being is seen as a wide phenomenon affecting personal well‐being and satisfaction in work and the community as a whole (Laine et al., 2018a, 2018b; Pahkin, 2015; Saaranen et al., 2015). The theoretical framework of this research is the resource‐based Content Model for the Promotion of School Community Staff's Occupational Well‐being (Saaranen et al., 2007, 2015). The content model for occupational well‐being consists of four aspects: worker and work (e.g. mental and physical workload and health), working conditions (e.g. physical, chemical and biological factors), professional competence (e.g. competence and continuing education) and work community (e.g. management, work organization and social support; Saaranen et al., 2015; Figure 1). These four aspects contain factors that may promote or prevent occupational well‐being, depending on the resource and workload factors (Saaranen et al., 2018). Saaranen et al. (2007) and (Laine, Saaranen, et al., 2018; Laine, Tossavainen, et al., 2018) found that the work community appears to be more relevant than the other three aspects to occupational well‐being.

Plenty of research has been done on educators' well‐being (e.g. Chan et al., 2021; Hatzichristou et al., 2021; Hilger et al., 2021; Laine et al., 2018a, 2018b), and only a few studies have been conducted on the well‐being of educators working in the field of social and health care, focusing on job satisfaction (Arian et al., 2018; Lee et al., 2017; Westphal et al., 2016; Worthy et al., 2020), professional competence (Hyvärinen et al., 2017), work community (Saaranen et al., 2021), occupational stress (Singh et al., 2020), occupational burnout (Wu et al., 2021), job retention (Lee et al., 2017; Westphal et al., 2016), life balance (Owens, 2017b) and quality of life (Keener et al., 2021). Comparison of these studies' results is challenging, and they have used very different theoretical frameworks. In the work community, factors related to management, organization and interaction with management and colleagues seem to be important (Arian et al., 2018; Lee et al., 2017; Owens, 2017b; Singh et al., 2020). Organizational management factors, such as collaborative management and educators' participation in decision‐making, were related to educators' job satisfaction (Arian et al., 2018; Lee et al., 2017; Worthy et al., 2020) and the intent to stay at the job (Lee et al., 2017). A working culture that encourages educators' autonomy, teamwork and mentoring was also related to the job satisfaction of nurse educators (Arian et al., 2018), workplace justice and supervisor support to lower personal burnout among clinical nurse educators (Wu et al., 2021). Educators' professional identification has been found to be positively related to job satisfaction (Gui et al., 2014).

The studies also revealed negative findings. A competitive working environment, lack of support and social bullying were associated with nurse educators' occupational stress and burnout (Singh et al., 2020). In Owens (2017b) study, nursing educators experienced insufficient support from management, and inadequate support and appreciation from colleagues, and they experienced bullying and rude behaviour from colleagues. The pressure on educators over the students' success, changing technological systems and time management challenges reduced the experience of satisfaction and life balance (Owens, 2017b). In addition, role conflict and role ambiguity can decrease job satisfaction (Arian et al., 2018). In a study by Brett et al. (2014), nursing educators experienced a difference between the current and ideal work environment in terms of active listening, open communication, shared decision‐making and support.

Very few research articles have provided interventions to improve the occupational well‐being of social and health care educators. Baker (2010) found positive outcomes from the formalized orientation programme on nurse educators' teaching competency, job satisfaction and retention. Online learning tool increased knowledge about workplace wellness among health care educators, health care employees and students (Blake & Gartshore, 2016), and gratitude interventions improved job satisfaction (Stegen & Wankier, 2018). Beyond the field of social and health care education, interventions to maintain and promote educators' occupational well‐being have previously been implemented to promote mental and physical well‐being (Ohadomere & Ogamba, 2021; Rinne et al., 2021). Additional holistic occupational well‐being development has been implemented in various research and development projects, where the need to develop occupational well‐being emerges from the needs of the education organization and the staff is committed to implementing occupational well‐being activities (Laine et al., 2018a; Saaranen et al., 2015; Woynarowska‐Sołdan, 2019).

Some demographics, such as age, work experience, type of employment contract, and education organization, have been found to be related to educators' well‐being (Arian et al., 2018; Hyvärinen et al., 2017; Saaranen et al., 2021; Westphal et al., 2016). The connection between age and well‐being is contradictory. Age can reduce experiences from the functionality of the work community (Saaranen et al., 2021) and job satisfaction (Arian et al., 2018). However, in a study by Wu et al. (2021), younger educators experienced a higher risk of occupational burnout than older ones. On the contrary, work experience can increase job satisfaction (Arian et al., 2018). The short length of an annual employment contract can decrease job satisfaction (Arian et al., 2018). Also, in a previous Finnish study, health care educators from vocational institutions experienced a higher level of community occupational well‐being than educators from universities of applied sciences (Hyvärinen et al., 2017). The teaching profession can be seen as a very independent profession, where autonomy and work and working time flexibility increase job satisfaction (Singh et al., 2020; Westphal et al., 2016). Therefore, in addition to the previously mentioned demographics, the possibility of remote work can be considered to increase occupational well‐being.

When examining factors associated with occupational well‐being, employee background factors, organizational management and functionality of the work community should be taken into account (Arian et al., 2018; Laine et al., 2018a, 2018b; Saaranen et al., 2021). It is also important to distinguish experiences from the employees' personal and work community occupational well‐being because these experiences are often different (Hyvärinen et al., 2017; Saaranen et al., 2013, 2015), but only a few studies have addressed this issue in social and health care education (Hyvärinen et al., 2017; Saaranen et al., 2020, 2021). This is the first Finnish nation‐wide study on the occupational well‐being of the work community in social and health care education. The study was conducted during the COVID‐19 pandemic, when social, face‐to‐face connections and activities in work communities were unexpectedly reduced (Keener et al., 2021). The aim of this cross‐sectional study was to evaluate factors related to the occupational well‐being of social and health care educators' work communities during the COVID‐19 pandemic. By understanding the factors related to occupational well‐being in the work community during exceptional periods, it will also be easier to respond to needs in the future.

The research questions of this study include the following:

- What is the state of educators' personal and community occupational well‐being?
- How do educators experience the work community aspect of occupational well‐being (based on the work community subscales)?
- What factors are related to the work community subscales?
- What factors are related to community occupational well‐being?

## 3 METHODS

### 3.1 Design

This quantitative cross‐sectional study was conducted among social and health care educators in Finland during the COVID‐19 pandemic in autumn 2020. The study was reported according to STROBE guidelines.

### 3.2 Data collection

The target group included social and health care educators working in universities of applied sciences and vocational institutions and members of the Vocational Educators and Trainers association (Ammatilliset opettajat AO ry; *N* = 1772) in Finland. The association includes about 70% of all social and health care educators, which represents a sufficient share of the study population. The data were collected by electronic survey (Webropol) in autumn 2020 via the contact person from the Vocational Educators and Trainers association.

### 3.3 Instrument

The research uses a part of the Finnish Occupational Well‐Being of Social and Health Care Teachers Index Questionnaire, which is based on Saaranen et al., 2006 Well‐Being at Your Work Index Questionnaire. The original questionnaire was modified and adapted in 2011, 2017 (Hyvärinen et al., 2017; Saaranen et al., 2020), and again in 2020 to measure social and health care educators' occupational well‐being.

The questionnaire contains 104 variables, of which the following are used in this study. Personal and community occupational well‐being and satisfaction with the activities promoting occupational well‐being provided by the employer in the work community and self‐motivated occupational well‐being activities during leisure time were measured by four continuous variables (scale 0–5, 0 = very poor, 5 = very good). The work community aspect of the questionnaire contains 23 Likert scale variables (1 = totally disagree, 5 = totally agree). In addition, the following background variables were requested: education organization (vocational institution/university of applied sciences), degree level (bachelor/master/doctor), employment contract (permanent/temporary), remote work (yes/no), age (years) and work experience (years).

### 3.4 Data analysis

The data were analysed using IBM SPSS statistics 27. Descriptive, exploratory factor analysis (EFA) and multiple regression modelling were used for analysing the data. The adequate sample size for EFA (500 as very good) and the regression modelling (10 cases per every predictor) was well exceeded (Field, 2018; Mundfrom et al., 2005). EFA with maximum likelihood extraction and varimax rotation was used to form the work community subscales (mean variables) from variables measuring the work community aspect of occupational well‐being. Two variables were excluded based on low correlations, communality and factor loadings. The remaining 21 variables were statistically significantly correlated mostly between 0.2 and 0.7, and the sampling adequacy and correlation matrix were suitable for factor analysis (Kaiser‐Meyer‐Olkin (KMO): 0.937; Bartlett's Test of Sphericity: *p* < 0.001). Based on the scree plot, variables were loaded on four factors, which explained 65% of the variance in the variables. The final factor structure was confirmed by Cronbach's alpha reliability analysis (0.779–0.887), and it was found to be good in all factors. (Field, 2018.) Based on EFA, four mean variables were formed and named as the work community subscales: Appreciation (three variables), Collegiality and work atmosphere (seven variables), Work arrangements (five variables) and Management and information (six variables).

The factors associated with the work community subscales and community occupational well‐being were examined by multiple regression modelling (forced entry). Intercorrelations between continuous variables entered into the models were examined by Pearson correlation, and intercorrelations between dichotomous and continuous variables were examined by point‐biserial correlation. In the first set of models, connections between independent variables (background variables, satisfaction with occupational well‐being promoting activities in the work community, and leisure time, and personal occupational well‐being) and dependent variables (work community subscales) were examined separately. In the second model, connections between the same independent variables, with the addition of the mean variable (work community total scale) and the dependent variable (community occupational well‐being), were examined. The work community subscales were summed up as a single mean variable (work community total scale) to avoid possible multicollinearity between subscales. Categorical background variables (education organization, remote work, and employment contract) were dummy coded to fit the models. Based on the model diagnostics, the assumptions of the regression model appeared to be in order: linearity, no multicollinearity, homoscedasticity and normality of residuals. Missing data were deleted from the analysis by listwise deletion. Four error observations were changed as missing values (Field, 2018). The independent variables were chosen based on previous research findings (Arian et al., 2018; Hyvärinen et al., 2017; Laine et al., 2018a, 2018b; Saaranen et al., 2021; Westphal et al., 2016). After the analysis, 21 Likert scale variables from 1 to 5 were rescaled as 1 to 3 (1–2 = disagree, 3 = neither agree nor disagree, 4–5 = agree) to summarize the report.

### 3.5 Ethics

The study followed the responsible conduct of research recognized by the scientific community. Participation in this study was based on informed consent (Finnish National Board on Research Integrity, 2019). In spring 2020, the UEF Committee of Research Ethics (10/2020 12.6.2020) issued an ethical statement, and the Vocational Educators and Trainers in Finland association issued research approval for the collection of data. This study followed the General Data Protection Regulation (GDPR, 2016) with regards to the collecting, processing and storing of data.

## 4 RESULTS

### 4.1 Background information and personal and community occupational well‐being

A total of 552 educators in the social and health care fields participated in the study (participation rate of 31%). The average age of participants was 51 (SD 8.4), and ages ranged from 30 to 67 years. Most had a master's degree (88%), their education field was health care or both (health care and social services; 89%) and they worked as a lecturer (74%) in a permanent contract (91%). Educators had been working in the current workplace from 0 to 38 years (mean 12.2, SD 8.1). The mean of personal occupational well‐being was 3.2 (SD 1.13), and that of community occupational well‐being was 2.6 (SD 0.95). The mean of the satisfaction with the activities promoting occupational well‐being provided by the employer was 2.3 (SD 1.30), and that of self‐motivated occupational well‐being activities during leisure time was higher (mean 3.3, SD 1.06; Table 1).

**TABLE 1:** Demographics, occupational well‐being and activities promoting occupational well‐being.

Variable	*N* (%)	Mean (SD)
Age (*n* = 547)		51.2 (8.35)
Work experience in current workplace (*n* = 549)		12.2 (8.06)
Education organization (*n* = 552)		
Vocational institution	318 (57.8)	
University of applied sciences	232 (42.2)	
Education field (*n* = 551)		
Both (health care and social service)	247 (44.8)	
Health care	242 (43.9)	
Social service	62 (11.3)	
Remote work (*n* = 548)		
Yes	308 (56.2)	
No	240 (43.8)	
Employment contract (*n* = 548)		
Permanent	499 (91.1)	
Temporary	49 (8.9)	
^a^Occupational well‐being		
Personal occupational well‐being (*n* = 537)		3.19 (1.13)
Community occupational well‐being (*n* = 532)		2.62 (0.95)
^a^Activities maintaining and promoting occupational well‐being		
Occupational well‐being activities in work community (*n* = 494)		2.28 (1.30)
Occupational well‐being activities during leisure time (*n* = 521)		3.31 (1.06)

*Note*: *N* = Frequency, SD = standard deviation.

^a^ Scale 0–5, 0 = very poor, 5 = very good.

### 4.2 Educators' experiences of work community subscales

Appreciation (mean 3.88, SD 0.84) was the highest, and management and information (mean 2.90, SD 0.98) was the lowest of the work community subscales. Most (83%) educators regarded their work in the working community as important and meaningful, and over two‐thirds (70%) thought they were appreciated as an employee in the working community. Most (82%) felt that they receive help and support from their colleagues in challenging situations. Almost half of the educators (48%) experienced unequal treatment, and 20% disagreed that there is not bullying in the working community. Half of the educators experienced insufficient information about changes (51%) and were not satisfied with the organization of work (54%). Only 16% thought they were rewarded for a job well done in their work community (Table 2).

**TABLE 2:** Work community subscales, mean (scale 1–5), standard deviation (*SD*), percentage.

	Mean (SD)	Disagree %	Neither agree nor disagree %	Agree %
Appreciation (α 0.871) (*n* = 542)	3.88 (0.84)			
I regard my own work in the working community as important and meaningful		5.3	11.6	83.2
I am appreciated as an employee in my working community		11.3	18.9	69.8
My work is appreciated in my working community		12.3	21.0	66.7
Collegiality and work atmosphere (*α* 0.874) (*n* = 541)	3.36 (0.84)			
If necessary, I get help and support from my colleagues when facing challenging situations		8.2	10.4	81.5
There is trust in others' work input in my working community		16.1	19.4	64.5
There is no bullying in my working community		20.4	20.9	58.7
The interpersonal relationships between the employees at my workplace are fine		23.6	18.1	58.3
There is sufficient collaboration in my working community		36.1	17.5	46.4
In my working community, people can openly discuss things related to work		38.1	15.8	46.1
All educators are treated equally in my work community		48.2	17.1	34.7
Work arrangements (*α* 0.779) (*n* = 542)	2.99 (0.88)			
There is an opportunity for collaborative teaching in my working community		27.8	16.2	56.0
I am satisfied with my working time arrangements		37.3	14.0	48.7
I am satisfied with my opportunities to influence my working community		36.4	17.7	45.9
I am satisfied with the organization of work in my working community		54.2	14.4	31.5
Orientating new workers with their job and the working community has been successful		47.4	22.5	30.1
Management and information (*α* 0.887) (*n* = 546)	2.90 (0.98)			
My closest supervisor gives me help and feedback when I need it		33.9	18.1	48.0
The number of meetings we have in my working community is appropriate		35.8	17.1	47.2
The relationships between employees and their direct supervisors are working well at my workplace		34.3	19.4	46.3
My closest supervisor gives me enough information about the expectations concerning my work performance		36.6	18.0	45.4
Sufficient information has been provided about changes in the working community		50.8	16.9	32.3
I am rewarded for a job well done/my performance in my work community		61.2	22.6	16.1

*Note*: *α* = Cronbach's alpha.

### 4.3 Factors related to the work community subscales

Table 3 described the intercorrelations of variables entered in the multiple regression models.

**TABLE 3:** Correlations between background variables, work community subscales and variables measuring occupational well‐being and satisfaction with occupational well‐being activities.

	Age	WEX	RW^a^	EC^a^	EO^a^	APP	CWA	WAR	MIN	WCto	OWAwc	OWAlt	POW
Background													
WEX	0.620***												
RW^1^	−0.050	−0.061											
EC^1^	0.214***	0.344***	0.038										
EO^1^	−0.026	0.032	0.480***	0.009									
Work community													
APP	−0.126**	−0.100*	0.077	−0.068	−0.014								
CWA	−0.121**	−0.049	0.012	−0.073	−0.040	0.638***							
WAR	−0.152***	−0.121**	0.124**	−0.098*	0.078	0.524***	0.612***						
MIN	−0.126**	−0.115**	0.088*	−0.090*	0.037	0.531***	0.658***	0.723***					
WCto	−0.160***	−0.117**	0.077	−0.100*	0.006	0.792***	0.860***	0.851***	0.876***				
OW activities													
OWAwc	−0.058	−0.091*	0.000	−0.035	0.009	0.379***	0.491***	0.538***	0.605***	0.597***			
OWAlt	0.053	−0.007	0.075	−0.022	0.016	0.286***	0.208***	0.276***	0.180***	0.267***	0.311***		
OW													
POW	−0.109*	−0.114**	0.144**	−0.056	0.057	0.484***	0.459***	0.622***	0.512***	0.619***	0.529***	0.499***	
COW	−0.061	−0.116**	0.100*	−0.058	0.091*	0.400***	0.447***	0.620***	0.526***	0.611***	0.543***	0.311***	0.704***

*Note*: Significance level *p < 0.05; **p < 0.01; ***p < 0.001.

Abbreviations: APP, appreciation; COW, community occupational well‐being; CWA, collegiality and work atmosphere; EC, employment contract (permanent); EO, education organization (university of applied sciences); MIN, management and information; OW, occupational well‐being; OWAlt, occupational well‐being activities during leisure time; OWAwc, occupational well‐being activities in work community; POW, personal occupational well‐being; RW, Remote work (yes); WAR, work arrangements; WCto, work community total scale; WEX, work experience.

^a^ Point‐biserial correlation.

Age had a negative relationship with Appreciation, Collegiality and work atmosphere, and Work arrangements. Work experience in the current workplace had a positive relationship with Collegiality and work atmosphere. Satisfaction with the activities promoting occupational well‐being provided by the employer in the work community and personal occupational well‐being had a positive relationship with all work community subscales (*p* < 0.001) and best explained all work community subscales in the models, whereas the self‐motivated occupational well‐being activities during leisure time remained insignificant. The relationships between categorical background variables (remote work, type of education organization and employment contract) and work community subscales remained insignificant in all models (Table 4).

**TABLE 4:** Relationship of factors with work community subscales. Work community subscales as dependent variables.

	Work community subscales											
	Appreciation *R* ^2^ = 0.291			Collegiality and work atmosphere *R* ^2^ = 0.308			Work arrangements *R* ^2^ = 0.444			Management and information *R* ^2^ = 0.429		
Variables	*B* (SE)	*β*	CI	*B* (SE)	*β*	95% CI	*B* (SE)	*β*	95% CI	*B* (SE)	*β*	CI
Constant	3.319*** (0.269)		2.791 to 3847	3.065*** (0.274)		2.526 to 3.603	2.003*** (0.254)		1.503 to 2.502	2.167*** (0.287)		1.603 to 2.732
Age (years)	−0.012* (0.005)	−0.124	−0.022 to 0.002	−0.015** (0.005)	−0.153	−0.025 to to 0.005	−0.010* (0.005)	−0.101	−0.020 to −0.001	−0.009 (0.005)	−0.080	−0.019 to 0.001
Work experience	0.008 (0.005)	0.077	−0.003 to 0.019	0.013* (0.006)	0.126	0.003 to 0.024	0.004 (0.005)	0.033	−0.006 to 0.014	0.003 (0.006)	0.027	−0.008 to 0.015
Remote Working	0.046 (0.076)	0.028	−0.103 to 0.196	0.022 (0.077)	0.013	−0.130 to 0.175	0.054 (0.071)	0.031	−0.086 to 0.194	0.046 (0.080)	0.024	−0.112 to 0.204
Employment contract	−0.116 (0.120)	−0.041	−0.353 to 0.120	−0.180 (0.123)	−0.062	−0.420 to 0.061	−0.109 (0.114)	−0.037	−0.332 to 0.114	−0.185 (0.128)	−0.055	−0.436 to 0.067
Education organization	−0.130 (0.075)	−0.080	−0.277 to 0.016	−0.103 (0.076)	−0.061	−0.252 to 0.046	0.033 (0.070)	0.019	−0.104 to 0.171	0.021 (0.079)	0.011	−0.134 to 0.176
OW activities working community	0.117*** (0.030)	0.186	0.058 to 0.175	0.227*** (0.030)	0.350	0.167 to 0.287	0.199*** (0.028)	0.297	0.144 to 0.255	0.351*** (0.032)	0.472	0.289 to 0.413
OW activities leisure time	0.037 (0.035)	0.048	−0.032 to 0.106	0.000 (0.036)	−0.001	−0.071 to 0.070	0.011 (0.033)	0.014	−0.054 to 0.076	−0.071 (0.037)	−0.078	−0.144 to 0.003
Personal OW	0.271*** (0.037)	0.372	0.197 to 0.344	0.196*** (0.039)	0.260	0.120 to 0.272	0.335*** (0.035)	0.431	0.266 to 0.405	0.248*** (0.040)	0.286	0.170 to 0.326

*Note*: Significance level *p < 0.05; **p < 0.01; ***p < 0.001.

Abbreviations: *β*, standardized coefficients beta; *B*, Unstandardized coefficients; CI, 95% confidence interval for *B*; SE, standard error; OW, occupational well‐being.

### 4.4 Factors related to community occupational well‐being

Age was the only background variable to have a positive relationship with community occupational well‐being. Personal occupational well‐being, activities promoting occupational well‐being provided by the employer in the work community, and work community total scale had strong positive connections to community occupational well‐being. Connection between self‐motivated occupational well‐being activities during leisure time and community occupational well‐being remained insignificant. Personal occupational well‐being best explained community occupational well‐being in the model. The model accounted for 57% of the variation in community occupational well‐being (Table 5).

**TABLE 5:** Relationship of factors and work community total scale with community occupational well‐being. Community occupational well‐being as dependent variables.

	*B* (SE)	*β*	CI	*p* Value
Model *R* ^2^ = 0.573				
Constant	−0.324 (0.298)		−0.910–0.261	0.277
Age (years)	0.010 (0.005)	0.089	0.001–0.019	0.031
Work experience	−0.010 (0.005)	−0.080	−0.020–0.000	0.062
Remote Working	−0.091 (0.072)	−0.047	−0.232–0.050	0.204
Employment contract	−0.010 (0.111)	−0.003	−0.228–0.208	0.927
Education organization	0.129 (0.070)	0.067	−0.010–0.267	0.068
OW activities work community	0.104 (0.031)	0.140	0.042–0.166	0.001
OW activities leisure time	0.012 (0.033)	0.013	−0.054–0.077	0.727
Work community total scale	0.262 (0.059)	0.202	0.147–0.378	0.000
Personal OW	0.443 (0.041)	0.509	0.363–0.523	0.000

Abbreviations: *β*, standardized coefficients beta; *B*, unstandardized coefficients; CI, 95% confidence interval for *B*; SE, standard error.

## 5 DISCUSSION

This study evaluated factors related to the occupational well‐being of social and health care educators' work communities during the COVID‐19 pandemic. Community occupational well‐being was evaluated to be lower than personal occupational well‐being. Age was related to Work arrangements, Collegiality and work atmosphere, Appreciation and community occupational well‐being, whereas work experience was related only to Collegiality and work atmosphere. Personal occupational well‐being and activities promoting occupational well‐being provided by the employer in the work community are strong predictors of occupational well‐being on the work community level.

Previous studies have also found community occupational well‐being to be lower than personal occupational well‐being (Hyvärinen et al., 2017; Laine et al., 2018a), but the difference was considerable in this study. The COVID‐19 pandemic has decreased the interactions between employers (Keener et al., 2021) and could increase feelings of isolation among the work community. In this study, educators considered their work meaningful and experienced collegiality in terms of support and help, but unequal treatment and even bullying were experienced at the same time. Emotional support can be seen as recourse that helps to deal with challenges and stresses related to the COVID‐19 pandemic (Chan et al., 2021). Saaranen et al. (2021) also discovered experiences of bullying and unequal treatment in a Finnish study, but these kinds of experiences increased in this study. Also, in a study by Owens, 2017b nursing educators experienced bullying and rude behaviour from colleagues. Bullying can cause occupational stress and burnout and decrease the retention of staff (Singh et al., 2020). This study did not reveal who caused the bullying and unequal treatment, so it remains unclear whether it was management, employees or students. Experiences of bullying and unequal treatment of Finnish social and health care educators should be further explored.

Regarding the background variables, age and work experience were the only significant predictors of the work community subscales and community occupational well‐being. Interestingly, based on the negative slope, older educators experienced Work arrangements, Collegiality and work atmosphere, and Appreciation slightly less than younger educators, but the experience of community occupational well‐being was higher. Also, in Saaranen et al. (2021) study, older educators felt that the functionality of the work community was lower than younger educators. There is some evidence that, prior to the COVID‐19 pandemic, older educators did not give a high value to the development of technological skills (Kotcherlakota et al., 2017). Because of this, educators may have found the transition to e‐learning and related arrangements to be more challenging than younger educators. Furthermore, work experience in the current workplace increased Collegiality and work atmosphere, whereas age reduced it. Based on the slope in the model, the connection between age and Collegiality and work atmosphere remains rather small because work experience reduces the association. However, it should be noted that the age of a vocational teacher does not directly indicate the extent of work experience. In a systematic review, Arian et al. (2018) found evidence that age reduces job satisfaction and that work experience increases it.

Based on this study, personal occupational well‐being and activities promoting occupational well‐being provided by the employer in the work community strongly predict community occupational well‐being and a higher experience of the work community subscales. There is some evidence that people with high personal occupational well‐being also rate their experience in the work community at a higher level (Perkiö‐Mäkelä et al., 2021). In this study, the activities promoting occupational well‐being provided by the employer were experienced at a rather low level, and this would also seem to give some reason for the low experience of community occupational well‐being. Work community total scale was one of the strongest predictors of community occupational well‐being in the model, indicating the importance of management, work arrangement and communal issues in promoting community occupational well‐being. Based on the correlation statistics, Work arrangements and Management and information had the strongest connection to community occupational well‐being. Previous studies have found that communal issues, such as working atmosphere and appreciation of others' work, have a larger relationship with community occupational well‐being (Laine et al., 2018b; Saaranen et al., 2007). It can be assumed that, during the COVID‐19 pandemic, work arrangements, information and management issues became more important to well‐being when work is done in challenging environments and constantly changing conditions. Support is needed for educators to manage properly in these conditions (Chan et al., 2021). In this study, many educators had a relatively low experience of information provision, opportunities to influence and rewards for a job well done. Collaborative management, shared decision‐making, equal pay and other rewarding practices are important when considering job satisfaction (Arian et al., 2018; Lee et al., 2017; Worthy et al., 2020) and the intent to stay at a job (Lee et al., 2017).

The surprising result was that although personal occupational well‐being was the strongest predictor of the work community subscales and community occupational well‐being, the self‐motivated occupational well‐being activities during leisure time remained an insignificant predictor in all models. However, this is supported by the fact that self‐motivated activities during leisure time focus on individual health and well‐being promotion and not so much on promoting well‐being on the community level. Based on this result, when promoting occupational well‐being in the work community, the importance of activities that promote occupational well‐being on the work community level should be emphasized, and the importance of one's own personal experience of occupational well‐being for the work community. The finding also supports the needs for occupational well‐being actions related to the work community aspect, such as management, information provision, work organization and rewarding practices, and communal issues, such as unequal treatment and bullying. However, limited research has been done on the interventions to promote occupational well‐being in the work community in social and health care education (Baker, 2010; Blake & Gartshore, 2016; Stegen & Wankier, 2018). When promoting occupational well‐being on the work community level, the development activities should be based on the identified needs from the work community that ensure continual development (Laine et al., 2018a; Saaranen et al., 2015; Woynarowska‐Sołdan, 2019). Even in exceptional times, the importance of promoting occupational well‐being cannot be overlooked, but promoting activities must be developed to suit these exceptional circumstances.

### 5.1 Strengths and limitations of the study

The strength of this nationwide study was the large number of participants and the widely used questionnaire. Conducting the survey through a registered association made it possible to obtain respondents nationwide. The questionnaire used in this study, based on Saaranen et al., 2006 Well‐Being at Your Work Index Questionnaire, has been utilized in promoting the occupational well‐being of school staff nationally (Saaranen et al., 2006) and internationally (Laine et al., 2018a; Saaranen et al., 2015), and the modified questionnaire was piloted and pretested before this study.

The limitation of this study was the relatively low response rate and cross‐sectional study design. Despite the low response rate, the sample size was considered adequate for the analysis (Field, 2018; Mundfrom et al., 2005). However, it is possible that only those interested in occupational well‐being responded to the survey and that those who experienced low occupational well‐being and high workload and hurry left the survey unanswered. Predictors in this cross‐sectional study should be treated with caution, and confirmation would require repeating the analysis on new data. The results can be generalized in the context of Finnish social and health care education, but the results are not directly applicable to another time and context.

## 6 CONCLUSIONS

Based on this study, the experience of community occupational well‐being is lower than personal occupational well‐being. Educators' older age can be related to a lower experience of work arrangements, collegiality and work atmosphere, and appreciation, whereas a longer work experience to a higher experience of collegiality and work atmosphere. A high experience of personal occupational well‐being and employer‐provided occupational well‐being promotion activities increases experiences of work community and community occupational well‐being.

## 7 RECOMMENDATIONS

The activities that promote occupational well‐being on the work community level are important and should be targeted to the prevailing circumstances and understand the meaning of one's own personal experience of occupational well‐being for the work community. Actions promoting occupational well‐being should focus on management, information provision, work organization, rewarding activities and the equal treatment of employees. Special attention should be paid to improve work community issues for older educators. Further research is needed about community‐level occupational well‐being interventions focusing on management and information provision and identified development needs of the work community in social and health care education.

## AUTHOR CONTRIBUTIONS

The authors (Vauhkonen A, Saaranen T, Honkalampi K) have contributed to the design of the study and/or acquisition of data, and authors (Vauhkonen A, Saaranen T, Hujo M, Hult M, Honkalampi K) have contributed to the analysis and interpretation of data. All authors have drafted the article, read the final version and approved its publication.

## FUNDING INFORMATION

This research was supported by the OAJ's Occupational Wellbeing Fund, and the Finnish Nursing Education Foundation.

## CONFLICT OF INTEREST STATEMENT

The authors declared no potential conflicts of interest with respect to the research, authorship and/or publication of this article.

## PATIENT OR PUBLIC CONTRIBUTION

Finnish educators, nation‐wide, contributed to the study as participants.

## Data Availability

Research data are not shared.
